# Clinical significance of HIF-1α, ET-1, and NO as biomarkers in chronic obstructive pulmonary disease patients with pulmonary hypertension

**DOI:** 10.17305/bb.2024.11078

**Published:** 2024-10-15

**Authors:** Xuan Zhang, Youxia Zhou, Huicong Zhang

**Affiliations:** 1 Respiratory Intensive Care Unit, Hebei Provincial Hospital of Traditional Chinese Medicine, Shijiazhuang, Hebei, China; 2 Department of General Internal Medicine, Shijiazhuang Fourth Hospital, Shijiazhuang, Hebei, China; 3 Respiratory Department, Hebei Provincial Hospital of Traditional Chinese Medicine, Shijiazhuang, Hebei, China

**Keywords:** Chronic obstructive pulmonary disease, pulmonary hypertension, hypoxia-inducible factor 1-alpha, endothelin-1, nitric oxide

## Abstract

Pulmonary hypertension (PH) is a common complication of chronic obstructive pulmonary disease (COPD), with repeated infections and airflow limitation leading to endothelial dysfunction. The objective of this study was to retrospectively investigate the clinical significance of serum hypoxia-inducible factor 1-alpha (HIF-1α), plasma endothelin-1 (ET-1), and nitric oxide (NO) as non-invasive blood biomarkers for diagnosing patients with acute exacerbation of COPD (AECOPD). A total of 230 AECOPD patients were assessed for serum HIF-1α, plasma ET-1, and NO levels using Doppler echocardiography and blood tests. Clinical characteristics, including age, COPD duration, and comorbidities, were compared between patients with and without PH. The diagnostic value was determined through receiver operating characteristic analysis. Pearson correlation and multivariate logistic analyses explored the correlation of biomarkers in AECOPD with PH. Serum HIF-1α, plasma ET-1, and NO levels showed significant differences between COPD patients with and without PH. The combination model (0.027 * HIF-1α + 0.028 * ET-1 - 0.049 * NO) demonstrated an area under the curve of 0.89, indicating superior diagnostic value compared to individual biomarkers. Multivariate logistic analysis identified smoking, higher Global Initiative for Chronic Obstructive Lung Disease grade, hypertension, and elevated levels of HIF-1α, ET-1, and NO as independent risk factors for AECOPD. Positive correlations were observed between serum HIF-1α and plasma ET-1 levels with pulmonary artery systolic pressure, while NO demonstrated a negative correlation. Serum HIF-1α, plasma ET-1, and NO were associated with AECOPD and PH, and detecting individual or combined levels of these biomarkers in the blood can predict COPD-related PH.

## Introduction

Chronic obstructive pulmonary disease (COPD) is a prevalent and debilitating respiratory condition characterized by persistent airflow limitation. One of the most significant and life-threatening complications of COPD is pulmonary hypertension (PH), which arises from a complex interplay of several pathological processes. Among these, endothelial dysfunction plays a pivotal role, often triggered by repeated infections and chronic airflow limitation inherent to COPD [1]. The persistent inflammatory state, coupled with recurrent injury and repair mechanisms, creates an environment conducive to pulmonary vascular remodeling—a key contributor to the development and progression of PH in COPD patients [2, 3]. Current research firmly establishes that both PH and right heart dysfunction are independent risk factors that substantially increase mortality in COPD patients [4]. Furthermore, secondary PH frequently develops early in the course of COPD, driven by oxygen-related changes, with pulmonary vascular remodeling playing a critical role as the disease advances [5]. Despite the severe implications of PH in COPD, early detection remains challenging due to the non-specific nature of PH symptoms and their variability based on disease severity [6]. Therefore, timely and accurate assessment of PH severity is essential for effective clinical intervention and management of COPD.

Right heart catheterization is the most accurate method for detecting PH, but its invasive nature limits widespread use [7]. Doppler echocardiography is commonly used to estimate pulmonary artery systolic pressure (PASP), yielding results comparable to those from right heart catheterization [8]. However, it is unsuitable for patients with segmental wall motion abnormalities and has limitations, such as angle dependence, load dependence, single-dimensional motion assessment, and heart size dependence. Consequently, PASP cannot be fully assessed via Doppler echocardiography alone. These challenges underscore the need for alternative diagnostic approaches. Combining PASP estimation with serum markers like hypoxia-inducible factor 1-alpha (HIF-1α), endothelin-1 (ET-1), and nitric oxide (NO) could improve diagnostic accuracy for PH, particularly in COPD patients, allowing earlier detection and intervention to reduce or delay PH onset. Combining echocardiography with relevant serum markers may lead to more accurate PH diagnoses.

ET-1 is the most potent vasoconstrictor produced by endothelial cells, exerting its effects by binding to ET-A or ET-B receptors in pulmonary blood vessels. It is the strongest endogenous vasoconstrictor identified to date [9]. Elevated ET-1 levels in PH patients have been shown to correlate positively with pulmonary vascular resistance, pulmonary capillary pressure, and pulmonary artery pressure. There is also a clear positive correlation between ET-1 levels and adverse outcomes in PH [10, 11]. Multiple studies have suggested that sustained or intermittent hypoxia can stimulate increased synthesis and release of ET-1 by endothelial cells. Additionally, the imbalance in the ratio of ET-1 to NO leads to endothelial dysfunction, playing a crucial role in PH formation. Decreased NO levels are positively correlated with increased pulmonary artery wall thickness. The application of NO or NO-producing vasodilators has been reported to inhibit endothelial proliferation and muscularization in pulmonary blood vessels, suggesting that NO plays a key inhibitory role in pulmonary vascular remodeling in hypoxic PH (HPH) [12]. Furthermore, studies on adult PH have found that low oxygen levels can induce the production of a nuclear protein called hypoxia-inducible factor 1 (HIF-1), which binds to target gene sites, promoting transcription and triggering cellular responses to hypoxia. HIF-1α is a crucial transcription factor in the body’s adaptation to hypoxia and a key regulator in the development of HPH and hypoxic pulmonary vascular remodeling (HPSR) [13, 14].

Given the significant correlations of these biomarkers with COPD pathogenesis, this study aimed to investigate the relationship between serum HIF-1α, plasma ET-1, and NO in patients with acute exacerbation of COPD (AECOPD) and the development of PH. The results of this study could support the use of these biomarkers to facilitate the diagnosis of AECOPD and further improve clinical outcomes.

## Methods

### Inclusion criteria

The study was approved by the ethics committee of Hebei Provincial Hospital of Traditional Chinese Medicine (#HBZY2021-KY-080-01). Study participants were individuals diagnosed with AECOPD in our respiratory department between 2021 and 2023. Informed consent was obtained from each participant. Patients met the diagnostic criteria for AECOPD as outlined in the 2013 revised version of the “Guidelines for the Diagnosis and Treatment of Chronic Obstructive Pulmonary Disease” established by the Chinese Medical Association Respiratory Branch. These criteria include an acute worsening of respiratory symptoms, such as dyspnea, cough, or purulent sputum, leading to a confirmed AECOPD diagnosis. The COPD diagnosis also followed the 2013 guidelines. PASP was calculated using Doppler echocardiography by measuring tricuspid regurgitation pressure, with PASP greater than 35 mmHg indicating PH. Inclusion criteria required participants to be aged 18 and above.

### Exclusion criteria

The following individuals were excluded: those with non-AECOPD, concurrent liver, urinary system, or blood disorders, or tumors; individuals with respiratory diseases, such as asthma, interstitial lung disease, pneumonia, tuberculosis, lung cancer, or pulmonary embolism; and patients with heart failure, acute myocardial infarction, acute cerebral infarction, or acute cerebral hemorrhage.

### Pulmonary function classification

Pulmonary function was classified according to the standards set forth in the “Chinese Expert Consensus on Diagnosis and Treatment of Acute Exacerbation of Chronic Obstructive Pulmonary Disease.” Patients were categorized based on the ratio of forced expiratory volume in the first second (FEV1) to forced vital capacity (FVC) after bronchodilator inhalation. Airflow limitation was defined as FEV1/FVC < 70%, and severity was classified into four levels: Grade I (mild) with FEV1% ≥ 80%, Grade II (moderate) with 50% ≤ FEV1% < 79%, Grade III (severe) with 30% ≤ FEV1% < 50%, and Grade IV (very severe) with FEV1% < 30%. FEV1 and FVC were measured using the Masterscope spirometer (Jaeger, Germany), based on the Global Initiative for Chronic Obstructive Lung Disease (GOLD) standards.

**Figure 1.**
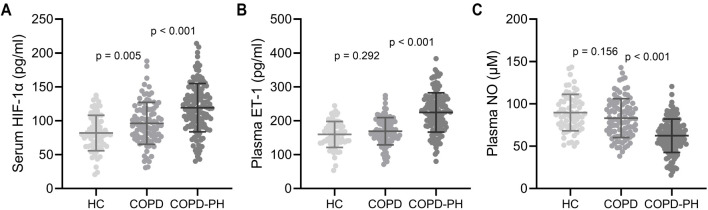
**Comparisons of serum HIF-1α (A), plasma ET-1 (B), and plasma NO (C) among healthy control (HC, *n* ═ 70), acute exacerbated COPD patients with (*n* ═ 136) or without (*n* ═ 94) PH.** The data were shown with mean ± SD. *P* values were calculated from the unpaired *t*-test with Welch’s correction. COPD: Chronic obstructive pulmonary disease; PH: Pulmonary hypertension; HIF-1α: Hypoxia-inducible factor 1-alpha; ET-1: Endothelin-1; NO: Nitric oxide.

In patients with AECOPD, whether they also had PH (Yes ═ 1, No ═ 0) was set as the dependent variable. Variables with significant differences in Table 1 and Figure 1 were used as independent variables. These independent variables were assigned values as follows: COPD course > 10 years: 1, otherwise 0; smoking: 1, otherwise 0; GOLD stage III-IV: 1, otherwise 0; hypertension: 1, otherwise 0; coronary heart disease: 1, otherwise 0; serum HIF-1α > 102.9 pg/mL: 1, otherwise 0; plasma ET-1 > 213.6 pg/mL: 1, otherwise 0; and plasma NO < 74.14 µM: 1, otherwise 0.

**Table 1:** Clinical characteristics of acute exacerbated COPD patients with or without PH

**Characteristics**	**COPD** **(*n* ═ 94)**	**COPD-PH (*n* ═ 136)**	***P* value**
Age (years)	63.48 ± 10.19	65.03 ± 11.82	0.226
COPD course (years)	9.24 ± 3.13	11.38 ± 4.06	0.008
*Gender*			
Male	67 (71.3%)	105 (77.2%)	0.355
Female	27 (28.7%)	31 (22.8%)	
*Smoke*			
Yes	36 (38.3%)	74 (54.4%)	0.022
No	58 (61.7%)	62 (45.6%)	
*GOLD*			
I-II	56 (59.6%)	52 (38.2%)	0.002
III-IV	38 (40.4%)	84 (61.8%)	
*Hypertension*			
Yes	25 (26.6%)	57 (41.9%)	0.018
No	69 (73.4%)	79 (58.1%)	
*Diabetes mellitus*			
Yes	18 (19.1%)	30 (22.1%)	0.624
No	76 (80.9%)	106 (77.9%)	
*Coronary heart disease*			
Yes	31 (32.9%)	65 (47.8%)	0.029
No	63 (67.1%)	71 (52.2%)	
*Hyperlipidemia*			
Yes	24 (25.5%)	43 (31.6%)	0.376
No	70 (74.5%)	93 (68.4%)	
Systolic pulmonary artery pressure (mmHg)	26.44 ± 3.71	60.21 ± 11.45	<0.001

The data are presented as mean ± SD or *n* (percentage). The comparisons of data were done by Mann–Whitney test or Unpaired *t*-test with Welch’s correction or Fisher’s exact test. GOLD: Global Initiative for Chronic Obstructive Lung Disease; COPD: Chronic obstructive pulmonary disease; PH: Pulmonary hypertension.

Multivariate logistic regression analysis was performed, showing that smoking, GOLD stage III-IV, concurrent hypertension, serum HIF-1α > 102.9 pg/mL, plasma ET-1 > 213.6 pg/mL, and NO < 74.14 µM were independent risk factors for PH in patients with AECOPD.

### Assessing pulmonary function and biomarker levels

During patient visits, pulmonary function was assessed using the Jaeger Masterscreen spirometer, and tricuspid regurgitation flow was measured using the Nemio 17 color Doppler ultrasound by experienced ultrasound technicians to estimate PASP. Additionally, 5 mL of venous blood was collected upon admission to measure serum HIF-1α and plasma ET-1 and NO levels.

### Statistical analysis

In the statistical analysis, the demographic and clinical characteristics of the study population were compared between COPD patients with and without PH. Group comparisons were conducted using the Mann–Whitney test, Unpaired *t*-test with Welch’s correction, or Fisher’s exact test. The diagnostic performance of serum HIF-1α, plasma ET-1, and NO, as well as their combined model, was evaluated through receiver operating characteristic (ROC) analysis. The area under the curve (AUC), along with sensitivity, specificity, and the Youden index, was reported for each biomarker and the combined model. Additionally, multivariate logistic regression analysis was performed to identify independent risk factors associated with AECOPD and PH. A *P* value of less than 0.05 was considered statistically significant, and data are presented as mean ± SD.

Fisher’s exact test and the chi-square test were used to analyze categorical data and assess associations between variables. Fisher’s exact test is particularly useful for small sample sizes and 2 × 2 tables, while the chi-square test is more suitable for larger datasets and can handle tables of various sizes.

## Results

### Clinical differences between group COPD and COPD-PH

This study included 230 eligible patients with AECOPD. PASP was calculated using Doppler echocardiography to measure tricuspid regurgitation pressure, with PASP greater than 35 mmHg considered as PH. The diagnosis revealed that 136 patients had PH (group COPD-PH), while 94 did not (group COPD). A comparison of basic clinical characteristics between the two groups, including age, COPD duration, gender, smoking status, GOLD classification, and the presence of hypertension, diabetes, coronary heart disease, and hyperlipidemia, showed significant differences in COPD duration (*P* ═ 0.008), smoking status (*P* ═ 0.022), GOLD classification (*P* ═ 0.002), hypertension (*P* ═ 0.018), and coronary heart disease (*P* ═ 0.029). In addition, serum HIF-1α, plasma ET-1, and NO levels were analyzed between the two groups. Significant up-regulation of HIF-1α (COPD: 96.17 ± 30.88 pg/mL, COPD-PH: 119.29 ± 35.69 pg/mL, *P* < 0.001, Figure 1A) and ET-1 (COPD: 169.02 ± 40.21 pg/mL, COPD-PH: 224.73 ± 57.99 pg/mL, *P* < 0.001, Figure 1B) levels, and significant down-regulation of NO (COPD: 83.15 ± 22.94 µM, COPD-PH: 62.38 ± 19.70 µM, *P* < 0.001, Figure 1C) were revealed in the COPD-PH group.

### Enzyme linked immunosorbent assay (ELISA)

Upon admission, 5 mL of venous blood was collected from each patient to measure serum HIF-1α, plasma ET-1, and NO levels. The blood samples were immediately processed by centrifugation at 3000 rpm for 10 min to separate serum and plasma. These were then aliquoted and stored at –80 ^∘^C until further analysis to prevent biomarker degradation. Concentrations of HIF-1α, ET-1, and NO were determined using ELISA kits, following the manufacturer’s instructions. All measurements were done in duplicate to ensure accuracy and reliability. Absorbance was read at the specified wavelength using a microplate reader, and biomarker concentrations were calculated based on standard curves generated for each assay.

**Table 2:** Diagnostic values in ROC analysis

	**Cut off**	**AUC**	**95% CI**	***P***	**Sensitivity (%)**	**Specificity (%)**	**Youden index**
Serum HIF-1α	102.9 pg/mL	0.69	0.63–0.76	<0.001	69.12	64.89	0.34
Plasma ET-1	213.6 pg/mL	0.78	0.72–0.84	<0.001	59.56	90.43	0.50
Plasma NO	74.14 µM	0.75	0.68–0.81	<0.001	74.26	61.70	0.36
Combination	-	0.89	0.85–0.93	<0.001	77.94	87.23	0.65

COMPUTE Combination ═ 0.027 * HIF-1α + 0.028 * ET-1 - 0.049 * NO. CI: Confidence interval; AUC: Area under the curve; ROC: Receiver operating characteristic; HIF-1α: Hypoxia-inducible factor 1-alpha; ET-1: Endothelin-1; NO: Nitric oxide.

**Figure 2.**
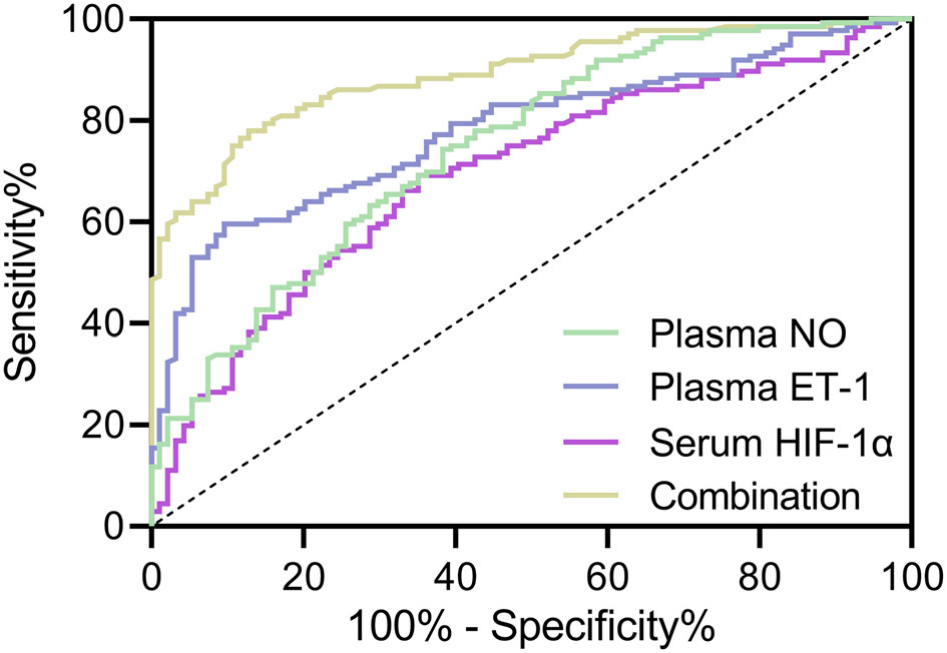
**ROC analysis of diagnostic values of serum HIF-1α, plasma ET-1, plasma NO, and their combination test for PH in acute exacerbated COPD patients.** ROC: Receiver operating characteristic; COPD: Chronic obstructive pulmonary disease; PH: Pulmonary hypertension; HIF-1α: Hypoxia-inducible factor 1-alpha; ET-1: Endothelin-1; NO: Nitric oxide.

### Diagnostic value of HIF-1**α**, ET-1, and NO

To explore the diagnostic value of serum HIF-1α, plasma ET-1, and NO levels (including their combination model of “COMPUTE Combination: 0.027 * HIF-1α + 0.028 * ET-1 − 0.049 * NO”) in AECOPD, ROC analysis was conducted. The results are shown in Figure 2 and Table 2, which include cut-off values, AUC, 95% CI, *P* value, sensitivity, specificity, and the Youden index. The sensitivity and specificity were determined using the maximum Youden Index, with AUC > 0.5 and *P* < 0.001 observed in all groups. Notably, plasma ET-1 and NO, as well as the combination model, had AUC values greater than 0.7. The highest specificity reached 90.43% in the plasma ET-1 group, while the highest sensitivity reached 77.94% in the combination model for diagnosing AECOPD. The maximum AUC of 0.89 (95% CI: 0.63–0.76) was found in the combination model of serum HIF-1α, plasma ET-1, and NO levels, suggesting the best diagnostic value and an improvement over individual markers like serum HIF-1α (AUC ═ 0.69, 95% CI: 0.63–0.76), plasma ET-1 (AUC ═ 0.78, 95% CI: 0.72–0.84), and NO (AUC ═ 0.75, 95% CI: 0.68–0.81).

### Correlations between HIF-1**α**, ET-1 NO, and AECOPD with PH

**Figure 3.**
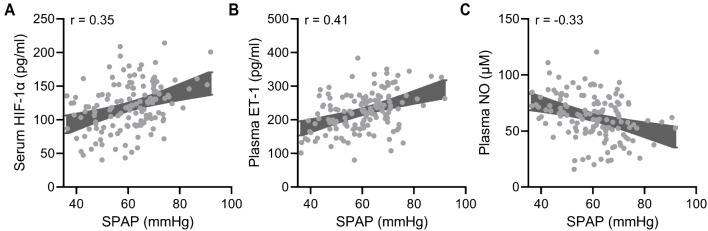
**Pearson correlation analysis of systolic pulmonary artery pressure with serum HIF-1α (A), plasma ET-1 (B), and plasma NO (C) in acute exacerbated COPD patients with pulmonary hypertension (*n* ═ 136).** *P* < 0.001 for all. COPD: Chronic obstructive pulmonary disease; HIF-1α: Hypoxia-inducible factor 1-alpha; ET-1: Endothelin-1; NO: Nitric oxide.

Since serum HIF-1α, plasma ET-1, and NO are potential biomarkers for predicting AECOPD, their correlations with AECOPD were further explored. Multivariate logistic analysis identified smoking (OR ═ 1.385, 95% CI ═ 1.229–1.671; *P* ═ 0.006), GOLD (OR ═ 1.558, 95% CI ═ 1.394–2.463; *P* < 0.001), hypertension (OR ═ 1.272, 95% CI ═ 1.097–1.459; *P* ═ 0.017), serum HIF-1α (OR ═ 1.058, 95% CI ═ 1.015–1.236; *P* ═ 0.026), plasma ET-1 (OR ═ 1.187, 95% CI ═ 1.048–1.334; *P* ═ 0.008), and NO (OR ═ 1.124, 95% CI ═ 1.065–1.409; *P* ═ 0.015) as independent risk factors for AECOPD (Table 3). Furthermore, correlation analysis demonstrated that serum HIF-1α (Figure 3A) and plasma ET-1 (Figure 3B) showed positive correlations with PASP, while NO (Figure 3C) showed a negative correlation.

**Table 3:** Multivariate logistic analysis for PH in exacerbated COPD patients

	**OR**	**95% CI**	***P* value**
COPD course > 10 years	1.113	0.938–1.316	0.121
Smoke	1.385	1.229–1.671	0.006
GOLD with III-IV	1.558	1.394–2.463	<0.001
Hypertension	1.272	1.097–1.459	0.017
Coronary heart disease	1.095	0.971–1.287	0.094
Serum HIF-1α > 102.9 pg/mL	1.058	1.015–1.236	0.026
Plasma ET-1 > 213.6 pg/mL	1.187	1.048–1.334	0.008
Plasma NO < 74.14 µM	1.124	1.065–1.409	0.015

CI: Confidence interval; GOLD: Global Initiative for Chronic Obstructive Lung Disease; COPD: Chronic obstructive pulmonary disease; PH: Pulmonary hypertension; HIF-1α: Hypoxia-inducible factor 1-alpha; ET-1: Endothelin-1; NO: Nitric oxide.

## Discussion

COPD is one of the most common chronic respiratory diseases, posing serious risks to human health. These risks stem not only from the progressive decline in lung function but also from a host of complications. PH in COPD is a well-established risk factor for poor outcomes, particularly in the later stages of the disease. The incidence of COPD with PH has been reported to range from 20% to 90%, highlighting the importance of early detection and intervention [15]. Unfortunately, the current GOLD standard for diagnosing PH is invasive and difficult to apply to most patients. Non-invasive echocardiography is widely used to estimate PASP, but it has its limitations. Patients with segmental wall motion abnormalities may not be eligible for echocardiography, and the method is also susceptible to interference from body movement and breathing. Additionally, echocardiography depends on factors, such as heart size, angle, and load, which prevent it from fully reflecting PASP [16]. The difficulty of performing echocardiography in critically ill patients further complicates diagnosis. Thus, combining blood biomarkers with diagnostic methods could offer a more accurate and early diagnosis, especially for patients with segmental wall motion abnormalities and those who are critically ill.

Hypoxia, endothelial dysfunction, and pulmonary vascular remodeling are key factors in the development of PH, a complication that increases mortality in COPD. Based on the pathological mechanisms involved, we screened three candidate biomarkers: ET-1, NO, and HIF-1α. ET-1, known for its vasoconstrictive effects, is associated with increased pulmonary vascular resistance and poor outcomes in PH patients. On the other hand, NO, a vasodilator, plays a critical role in preventing pulmonary vascular remodeling in hypoxic conditions. Hypoxia impacts the transcription and translation of endothelial NO synthase (eNOS) in vascular endothelial cells, reducing eNOS synthesis and leading to lower NO production [17]. This inability to produce sufficient NO limits its capacity to dilate blood vessels and counteract ET-1-induced vasoconstriction, contributing to increased PH [18]. Our data show opposing correlations of ET-1 and NO with PH, consistent with the previously reported imbalance between these molecules, which drives pulmonary vasoconstriction and induces PH. Furthermore, HIF-1α, a key transcription factor in the body’s response to hypoxia, plays a role in HPSR by regulating ET-1 transcription. Our results showed that both HIF-1α and ET-1 are positively correlated with PH, offering insights into the underlying mechanisms of PH in AECOPD.

The significance of this study is emphasized by the combination model, which demonstrated superior diagnostic value compared to individual biomarkers. ROC analysis showed that the combination of serum HIF-1α, plasma ET-1, and NO had an (AUC of 0.89, reflecting high diagnostic accuracy for AECOPD with PH. Additionally, the AUC values for plasma ET-1 and NO alone exceeded 0.7, indicating their substantial diagnostic potential. The high specificity and sensitivity of serum HIF-1α, plasma ET-1, NO, and the combination model further highlight their promise as reliable diagnostic tools for AECOPD. Multivariate logistic regression analysis also identified HIF-1α, ET-1, and NO as independent risk factors for AECOPD, reinforcing their importance in predicting COPD exacerbations and the development of PH. By identifying these blood biomarkers, clinicians can enhance early detection of PH in AECOPD patients, improving diagnosis and allowing for more timely interventions.

While this study provides valuable insights, it has some limitations. Its retrospective design and single-center cohort limit the generalizability of the findings. Future multicenter prospective studies are needed to validate these results and assess the utility of these biomarkers in various clinical settings. Additionally, it would be important to explore longitudinal changes in biomarker levels and their relationship to disease progression. Our study did not account for treatment factors during the stable phase of COPD, prior to acute exacerbations. Expanding research to include both AECOPD and stable COPD patients would further clarify the role of these biomarkers. Moreover, future studies should investigate the relationships between ET-1, NO, HIF-1α, common inflammatory biomarkers, and brain natriuretic peptide (BNP) levels to solidify their use in diagnosing AECOPD and better understand their mechanisms of action. Lastly, our findings, along with recent research into biomarkers [19], such as the neutrophil-to-lymphocyte ratio [20] and red cell distribution width [21], contribute to an expanding set of tools for diagnosing PH.

## Conclusion

In conclusion, our findings indicate the potentially predictive and diagnostic value of serum HIF-1α, plasma ET-1, and NO, along with their combined model, for AECOPD, particularly in patients with PH. This provides possibilities for early diagnosis and targeted interventions to reduce the mortality rate associated with COPD. The study highlights the clinical significance of serum HIF-1α [22], plasma ET-1 [23], and NO [24] in AECOPD patients with PH. The identified risk factors and the superior diagnostic value of the combination model underscore the potential utility of these biomarkers in clinical practice. Ultimately, this study enhances our understanding of the intricate relationship between these biomarkers and the development of PH in AECOPD, offering valuable insights for improved patient care and outcomes.
